# CT radiomic features for predicting resectability of oesophageal squamous cell carcinoma as given by feature analysis: a case control study

**DOI:** 10.1186/s40644-019-0254-0

**Published:** 2019-10-16

**Authors:** Jing Ou, Rui Li, Rui Zeng, Chang-qiang Wu, Yong Chen, Tian-wu Chen, Xiao-ming Zhang, Lan Wu, Yu Jiang, Jian-qiong Yang, Jin-ming Cao, Sun Tang, Meng-jie Tang, Jiani Hu

**Affiliations:** 1 Sichuan Key Laboratory of Medical Imaging, and Department of Radiology, Affiliated Hospital of North Sichuan Medical College, 63# Wenhua Road, Nanchong, 637000 Sichuan China; 2 Sichuan Key Laboratory of Medical Imaging, North Sichuan Medical College, Nanchong, 637000 Sichuan China; 3 Department of Radiology, Wayne State University, Detroit, MI USA

**Keywords:** Esophagus, Squamous cell carcinoma, Computed tomography, Esophagectomy, Diagnosis

## Abstract

**Background:**

Computed tomography (CT) is commonly used in all stages of oesophageal squamous cell carcinoma (SCC) management. Compared to basic CT features, CT radiomic features can objectively obtain more information about intratumour heterogeneity. Although CT radiomics has been proved useful for predicting treatment response to chemoradiotherapy in oesophageal cancer, the best way to use CT radiomic biomarkers as predictive markers for determining resectability of oesophageal SCC remains to be developed. This study aimed to develop CT radiomic features related to resectability of oesophageal SCC with five predictive models and to determine the most predictive model.

**Methods:**

Five hundred ninety-one patients with oesophageal SCC undergoing contrast-enhanced CT were enrolled in this study, and were composed by 270 resectable cases and 321 unresectable cases. Of the 270 resectable oesophageal SCCs, 91 cases were primary resectable tumours; and the remained 179 cases received neoadjuvant therapy after CT, shrank on therapy, and changed to resectable tumours. Four hundred thirteen oesophageal SCCs including 189 resectable cancers and 224 unresectable cancers were randomly allocated to the training cohort; and 178 oesophageal SCCs including 81 resectable tumours and 97 unresectable tumours were allocated to the validation group. Four hundred ninety-five radiomic features were extracted from CT data for identifying resectability of oesophageal SCC. Useful radiomic features were generated by dimension reduction using least absolute shrinkage and selection operator. The optimal radiomic features were chosen using multivariable logistic regression, random forest, support vector machine, X-Gradient boost and decision tree classifiers. Discriminating performance was assessed with area under receiver operating characteristic curve (AUC), accuracy and F-1score.

**Results:**

Eight radiomic features were selected to create radiomic models related to resectability of oesophageal SCC (*P-*values < 0.01 for both cohorts). Multivariable logistic regression model showed the best performance (AUC = 0.92 ± 0.04 and 0.87 ± 0.02, accuracy = 0.87 and 0.86, and F-1score = 0.93 and 0.86 in training and validation cohorts, respectively) in comparison with any other model (*P-*value < 0.001). Good calibration was observed for multivariable logistic regression model.

**Conclusion:**

CT radiomic models could help predict resectability of oesophageal SCC, and multivariable logistic regression model is the most predictive model.

## Background

Oesophageal cancer is the eighth most common malignant tumour worldwide [1]. The major histological type of this cancer is squamous cell carcinoma (SCC) [2]. At present, oesophagectomy is still the greatest curative treatment for patients with early-stage cancer (Stage T1 and T2). Patients with advanced oesophageal SCC (Stage T3 and T4a) may undergo neoadjuvant chemoradiotherapy before surgical resection. However, not all patients benefit from oesophagectomy. Patients with T4b-stage cancer or with distant metastases are regarded as having incurable disease, and these patients cannot undergo surgery but chemotherapy and/or radiotherapy [3, 4]. Like other malignant diseases, the option of the most suitable treatment has a remarkable effect on the prognosis of patients with oesophageal SCC. Therefore, it is crucial to determine resectability of oesophageal SCC for treatment decision making.

Computed tomography (CT) is commonly used in all stages of oesophageal SCC management including diagnosis, treatment guidance, and etc. [5]. The main role of CT at initial staging is to describe structural features of primary tumour, lymph node status and identification of metastasis. As reported, the accuracy of T staging and N staging with CT was 68 and 78%, respectively [5, 6]. However, the limitation of CT is to evaluate the intratumour heterogeneity of oesophageal SCC. In recent years, the attention of radiomics is increasing [7]. Its meaning is that high-through put extraction of large amount of information from images such as CT and magnetic resonance imaging enables tumour segmentation, feature extraction, and model establishment. With the help of exploration, prediction and analysis of massive image data information, physicians are assisted in making the most accurate assessment. Moreover, a set of multiple radiomic features is considered a more powerful diagnostic biomarker that can provide additional information for clinical data [8], and is reported to be an important predictor of distant metastasis, lymph node metastasis and preoperative staging based on the reports on lung cancer distant metastasis prediction [9], distant metastasis prediction of lymph nodes in colorectal cancer (CRC) [10] and preoperative CRC stage discrimination [11]. Although CT texture analysis has been applied and proved useful for predicting treatment response to chemoradiotherapy in oesophageal cancer [12, 13], the best way to use multiple imaging biomarkers as predictive markers for determining resectability of oesophageal SCC remains to be developed. To the best of our knowledge, there is no literature that has determined whether a CT radiomic model could enable the identification of resectability of oesophageal SCC. Therefore, the aim of this study was to develop CT radiomic features related to the identification of resectability of oesophageal SCC with multiple predictive models and to determine the most predictive model before individual treatment.

## Methods

### Patients

The retrospective study was approved by institution ethics committee. This study comprised an evaluation of the institutional database for medical records from January 2014 to December 2017 to identify patients with histologically biopsy-confirmed oesophageal SCC who underwent CT scans. According to the National Comprehensive Cancer Network (NCCN) based on CT scans [14], the criteria for unresectable oesophageal cancer were as follows: (1) cT4b tumours with involvement of the heart, great vessels, trachea, or adjacent organs including liver, pancreas, lung and spleen were considered unresectable; (2) oesophageal SCC with multi-station bulky lymphadenopathy was considered unresectable, although lymph node involvement should be considered in conjunction with other factors including age and performance status and response to therapy; or (3) oesophageal SCC with distant metastases including nonregional lymph nodes (stage IV) was unresectable. If the oesophageal SCC was not considered unresectable according to the NCCN guidelines, this tumour could be regarded resectable.

**Fig. 1:**
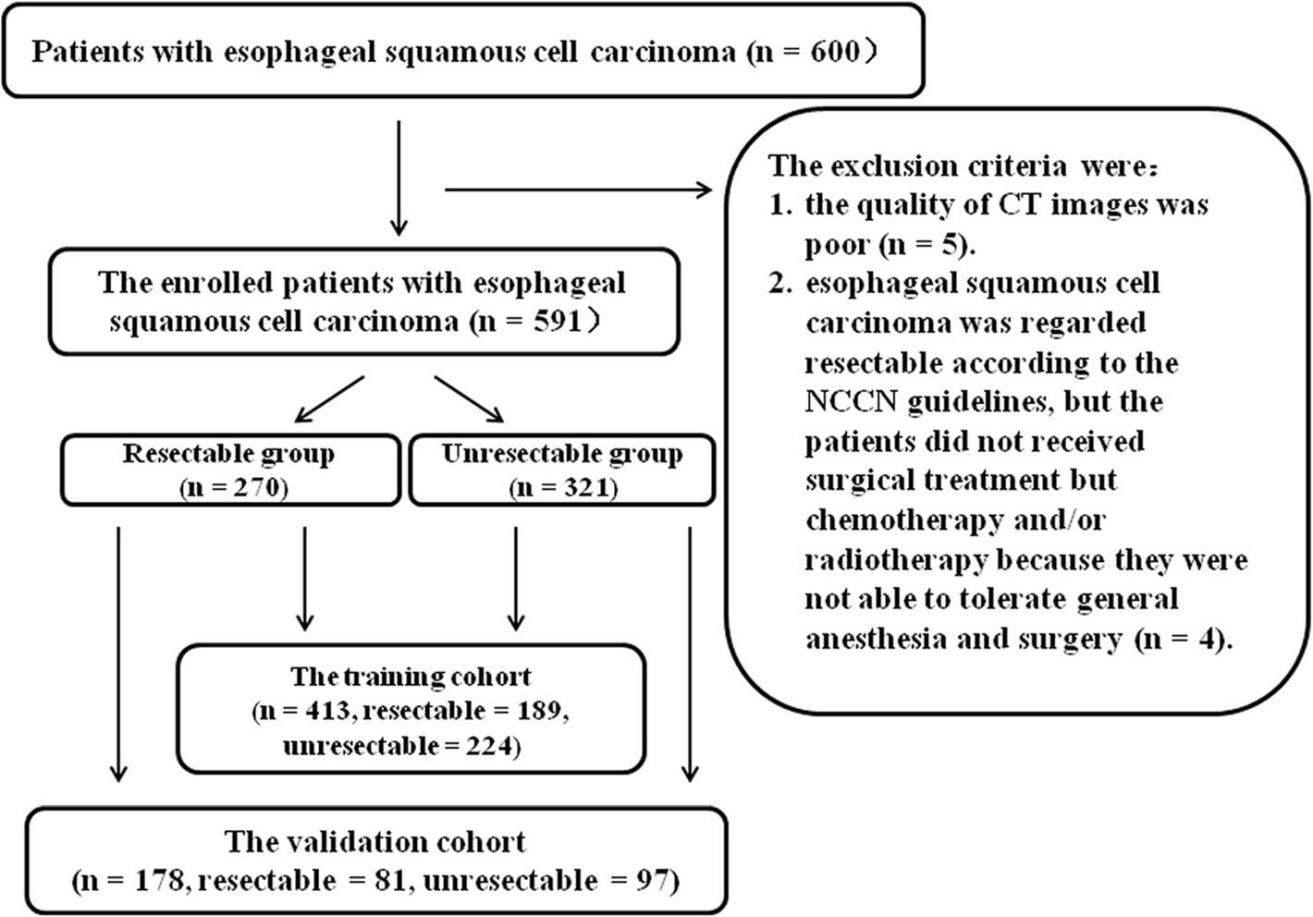
The CT data flow sequence in this research. Tumour contours are segmented manually by slice-by-slice delineating. In the training cohort, we select the extracted features depending on some rules. Based on the selected features, we build and validate the radiomic indicators. Ultimately, this research reveals that resectability of oesophageal squamous cell carcinoma is correlated with the radiomic indicators. LASSO, least absolute shrinkage and selection operator

Patients were enrolled into our study according to the following inclusion criteria: (a) the patients did not receive any tumour-related treatments (e.g., chemotherapy or radiotherapy) before undergoing CT for both resectable and unresectable oesophageal SCC groups; and (b) oesophageal SCC was regarded unresectable and resectable according to the previous NCCN guidelines based on CT findings. Totally, 600 consecutive patients with biopsy-confirmed oesophageal SCC were enrolled. The exclusion criteria were as follows: (a) the quality of CT images was poor (*n* = 5); or (b) oesophageal SCC was regarded resectable according to the previous NCCN guidelines, but the patients did not receive surgical treatment but chemotherapy and/or radiotherapy because they were not able to tolerate general anesthesia and surgery (*n* = 4). The patient flowchart is illustrated in Fig. 1. Of the previous 600 patients, 9 patients were excluded. Consequently, our study involved 591 cases (421 men and 170 women; mean age, 65.8 years; age range, 38–89 years). In the 591 enrolled patients, 270 and 321 patients had resectable and unresectable oesophageal SCC, respectively. Of the 270 patients with resectable oesophageal SCC, 91 patients with primary resectable tumours did not receive neoadjuvant therapy but surgery; and the remained 179 patients received neoadjuvant therapy after CT and before surgical treatment, the tumours shrank on therapy, the cases changed to resectable tumours, and these patients subsequently underwent successful surgery.

All patients were randomly allocated to the training and validation cohorts at the ratio of 7:3 based on the published report by Chen et al. [15]. Therefore, 413 oesophageal SCC patients were allocated to the training cohort, of whom 189 and 224 patients were in the resectable and unresectable subgroups, respectively. The remained 178 oesophageal SCC patients, including 81 in the resectable subgroup and 97 in the unresectable subgroup, were allocated to the validation group. Baseline clinical data including age, gender, tumour site, cigarette smoking, history of alcohol use and family history were derived from the medical records (Table 1). In the enrolled 591 patients, oesophageal SCC was histologically biopsy-confirmed. In all patients with resectable oesophageal SCC determined by the previous NCCN guidelines, the operation proved that all the cases could be resectable in both training and validation cohorts. All cases of unresectable oesophageal SCC met the criteria for unresectable oesophageal cancer according to the NCCN guidelines in both training and validation cohorts.

**Table 1:** Clinical characteristics of the training and validation cohorts

	The training cohort		The validation cohort	
	Resectable (*n* = 189)	Unresectable (*n* = 224)	Resectable (*n* = 81)	Unresectable (*n* = 97)
Median age (years)	52.8	65.8	58.8	62.6
Gender (%)				
Male	143 (75.6)	147 (65.6)	61 (75.3)	70 (72.1)
Female	46 (24.4)	77 (34.4)	20 (24.7)	27 (27.9)
Location of the tumour (%)				
Upper thoracic segment	14 (7.6)	13 (5.6)	4 (4.9)	5 (5.2)
Middle thoracic segment	135 (71.2)	155 (69.2)	60 (74.5)	50 (51.2)
Lower thoracic segment	40 (21.2)	56 (25.2)	17 (20.6)	42 (43.6)
Cigarette Smoking (%)				
Yes	115 (60.8)	154 (68.8)	59 (72.8)	66 (68.0)
No	74 (39.2)	70 (31.2)	22 (27.2)	31 (32.0)
History of alcohol use (%)				
Yes	94 (49.7)	134 (59.8)	51 (63.0)	63 (65.0)
No	95 (50.3)	90 (40.1)	30 (37.0)	34 (35.0)
Family History (%)				
Yes	85 (45.0)	150 (67.0)	35 (43.2)	58 (59.8)
No	104 (55.0)	74 (33.0)	46 (56.7)	39 (40.2)

### Image acquisitions

All patients underwent thoracic contrast-enhanced CT scans with two 64 multidetector scanners (LightSpeed VCT, GE Medical systems, USA). Before CT image acquisitions, 100- to 200-mL water was used as oral oesophageal negative contrast material. The image acquisitions were performed in the supine position. After a routine unenhanced scan, the contrast-enhanced CT data obtainment was started 25–30 s after the initiation of contrast agent (Omnipaque, Iohexol, GE Healthcare, USA) injection via a 20-G needle into an antecubital vein at a rate of 3 mL/s for a total of 70–100 mL tailored to body weight at the ratio of 1.5 ml/kg weight, followed by a 20-mL saline flush with a pump injector (Vistron CT Injection System, Medrad, USA) in order to show the enhanced features of this cancer. The CT scanning parameters in each patient were 120 kV_p_ of peak voltage, 200 mA of tube current (automatic exposure control employed), rotation time of 0.5 s, collimation of 64 × 0.6 mm, pitch of 0.9, slice thickness of 5 mm, and matrix of 512 × 512 mm. Examinations were performed during one breath-hold at full suspended inspiration for 10–15 s. The coverage of CT scan was from the neck to the middle of the left kidney. Subsequently, data were directly transferred to the General Electric Advantage Workstation 4.4 at the mediastinal window settings (window width, 400 HU; window level, 38 HU).

### Tumour segmentation and radiomic feature extraction

**Fig. 2:**
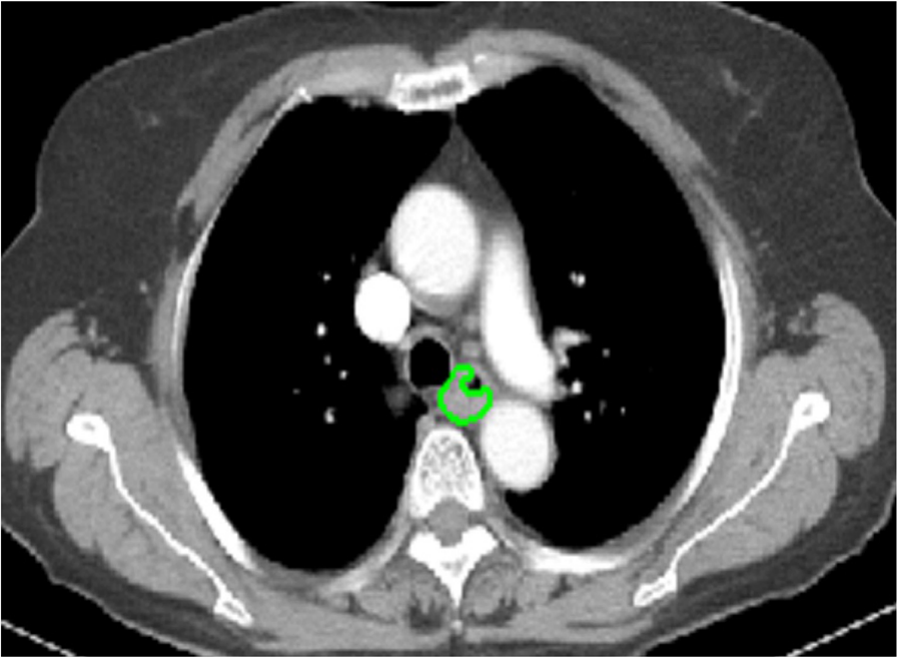
The tumour contours are segmented manually on thoracic contrast-enhanced CT image

The thoracic contrast-enhanced CT images with 5-mm thickness were imported into MATLAB 2016Ra for delineating the region of interest (ROI) of oesophageal SCC by using IBEX (β1.0, http://bit.ly/IBEX_MDAnderson) (Fig. 2) [16]. In our database, when the wall thickness was more than 5 mm on transverse images, the oesophageal wall was regarded abnormal for the delineation of tumoural ROI [17]. The primary three-dimensional (3D) ROI was manually delineated slice-by-slice in mediastinal window on the previous software package by two experienced radiologists (readers 1 and 2, with 2 and 21 years of clinical experience in digestive CT study interpretation, respectively). For each ROI, the contour of oesophageal SCC was drawn around the gross tumour volume avoiding air, fat and bone. The two radiologists reached a consensus by discussion when there were disagreements. When uncertainty concerning the tumour region existed, the area was not included in the ROI.

The ROI based texture feature extraction was performed with the MATLAB 2016Ra independently by the above-mentioned two experienced radiologists in a blinded fashion. A total of 495 radiomic features were extracted from the CT images for each oesophageal SCC to describe the tumour characteristics, and the 3D feature was obtained from the above 3D ROI based on intensity histogram, intensity direct, shape, gray-level co-occurrence matrix (GLCM), gray-level run-length matrix (GLRLM), and neighborhood gray-tone difference matrix (NGTDM) [18], which are detailed in Additional file 1.

### Dimensionality reduction and radiomic feature selection

The 495 previous resulting features were further processed to have zero mean and unit variance (z-score normalization) [19]:

$$ {x}_{norm}=\frac{x-\mu }{\sigma } $$

where x is the original feature value, μ is the mean value of this feature, and σ is the standard deviation.

To avoid the curse of dimensionality and reduce the bias from radiomic features when modeling, we adopted two steps to select the features in the training cohort. First, the least absolute shrinkage and selection operator (LASSO) method was used to identify the most useful predictive radiomic features for identifying resectability of oesophageal SCC because the LASSO regression model is suitable for the regression of high-dimensional data and enables to incorporate the panel of chosen features into a radiomic signature [20, 21]. The 1-standard error of the minimum criteria (the 1-SE criteria, a simpler model) was used to tune the regularization parameter (λ) and for feature selection using 10-fold cross-validation. Second, the features selected by the previous LASSO method were tested by the one-way ANOVA analysis to select potential important features for the training cohort. Features that did not meet either of the above tests were excluded.

### Construction of radiomic models

The optimal radiomic features created a predictive radiomic model based on the five machine learning methods including the multivariable logistic regression, decision tree, random forest, support vector machine (SVM), and X-Gradient boost. The radiomic model based on the selected radiomic features was developed using multivariable binary logistic regression with backward stepwise selection to build a linear classifier. The decision tree model described the tree structure that classified instances. Random forest was an important ensemble learning method based on Bagging, which could be used for classification, regression and other problems. A SVM with a Gaussian kernel was then used with the selected radiomic features to build a non-linear classifier. X-Gradient boost was a machine learning technique for regression and classification problems, which produced a prediction model in the form of an ensemble of weak prediction model.

The Hosmer-Lemeshow test was performed to test the reliability of calibration curves. A significant test implied that the model did not calibrate perfectly [22]. The calibration curve was used to evaluate the calibration of the most suitable model. The confusion matrix calculated the area under receiver operating characteristic curve (AUC), accuracy, F-1score to quantify the discrimination performance of the previous five models.

### Statistical analysis

Intraclass correlation coefficient (ICC) was used to quantify the intraobserver (reader 1 twice) and interobserver (reader 1 vs. reader 2) agreements of each of the 495 radiomic features extracted from the delineated ROIs in each patient. The radiomic features were considered to be reproducible when the ICC was greater than 0.75 [23].

LASSO regression was performed using the “glmnet” package of R software version 3.4.4 (http://www.Rproject.org) based on the multivariate binary logistic regression. The other analyses were performed using the “scikit-learn” packages of python 3.6 (http://www.python.org). The AUCs between the multivariable logistic regression and random forest, SVM, X-Gradient boost or decision tree model were compared using the ‘DeLong’ test. The reported statistical significance levels were all two-sided, and a *P* value less than 0.05 indicated statistical difference.

## Results

### Intra- and inter-observer variability assessment of feature extraction

The inter- and intra-observer reproducibility of the feature extraction was 0.76 to 1 for 483 features and less than 0.75 for 12 features. After this assessment, the 483 features (ICC ≥ 0.75) were selected from the 495 features. Thence, all results were derived from the measurements of reader 1.

### Feature selection and radiomic feature building

**Fig. 3:**
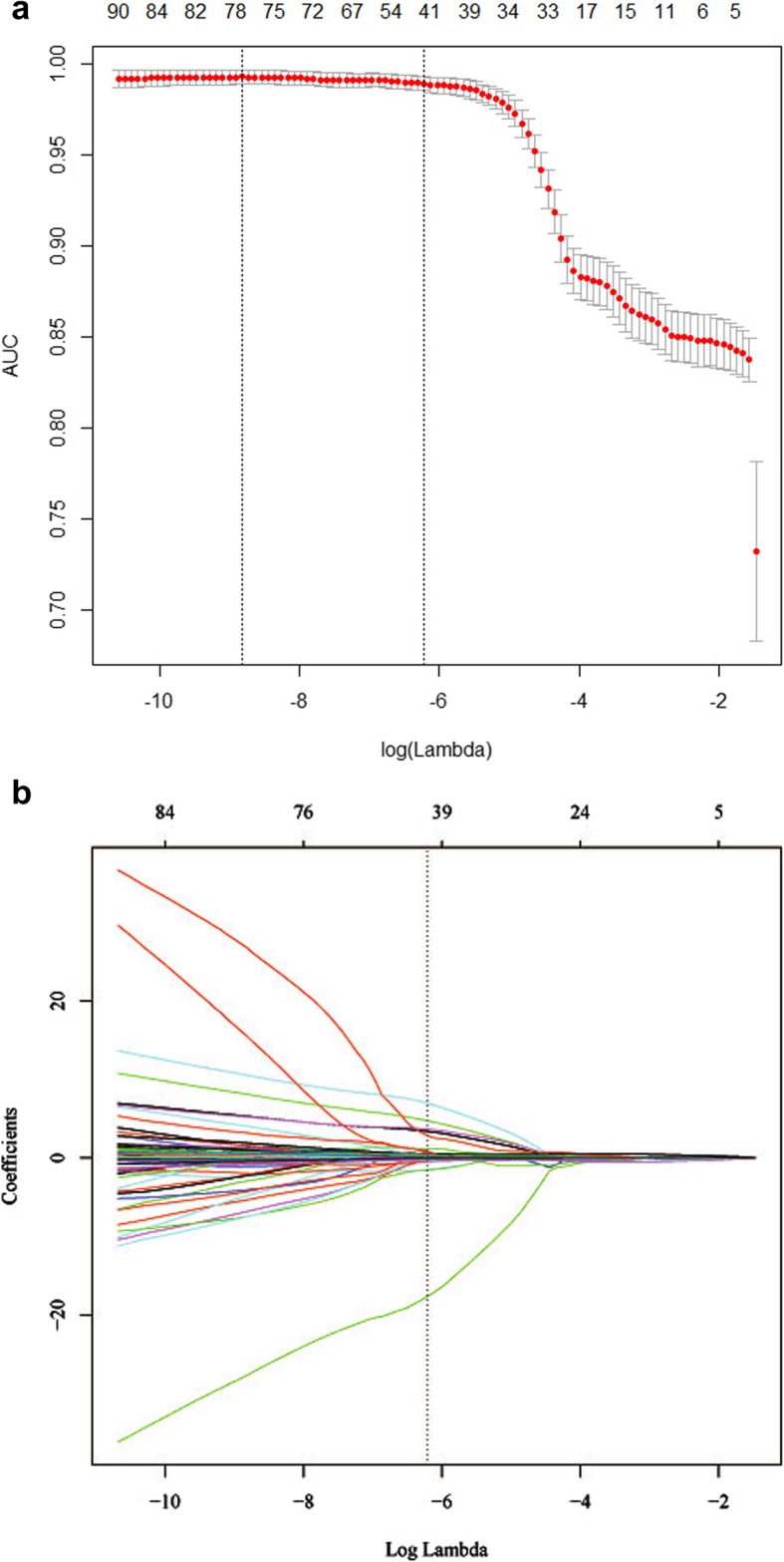
The least absolute shrinkage and selection operator (LASSO) binary logistic regression model used to select texture feature. **a** Tuning parameter (λ) selection in the LASSO model used 10-fold cross-validation via minimum criteria. The area under the receiver operating characteristic curve (AUC) is plotted versus log(λ). Dotted vertical lines are drawn at the optimal values by using the minimum criteria and the 1 standard error of the minimum criteria (the 1-SE criteria). log(λ) = −6.214, with λ chosen of 0.02. **b** LASSO coefficient profiles of the 483 texture features. A coefficient profile plot is produced against the log(λ) sequence. Vertical line is drawn at the value selected using 10-fold cross-validation, where optimal λ results in 42 non-zero coefficients

A total of 483 features were used for LASSO regression, and 42 features were selected by LASSO (11.5:1 ratio) (Fig. 3a and b). The process of selecting features with non-zero coefficients from the coefficient profiles was performed by using the optimized lambda (λ) of 0.02. Among the 42 features, the one-way ANOVA analysis showed that 8 features were significantly different (all *P*-values < 0.01). These features included two shape and size features, one intensity direct feature, and five texture features, which are detailed in Table 2.

**Table 2:** Selected features with descriptions

Feature	Mean of resectable SCC	Mean of unresectable SCC	*P-*value
X135.7Correlation	6.8	3.801	2.65 × 10^−11^
X45.1InverseVariance	11.32	15.317	< 2 × 10^−16^
X90.1InverseVariance	14.1	16.098	< 2 × 10^− 16^
X90.1MaxProbability	1.95	1.747	0.0006
Kurtosis	3.32	1.623	9.16 × 10^−7^
Coarseness	9.08	7.078	4.56 × 10^−13^
Convex	3.71	4.91	2.07 × 10^−5^
Orientation	4.2	2.4	0.0044

*SCC* squamous cell carcinoma

### Model building and predictive performance of the five models

**Fig. 4:**
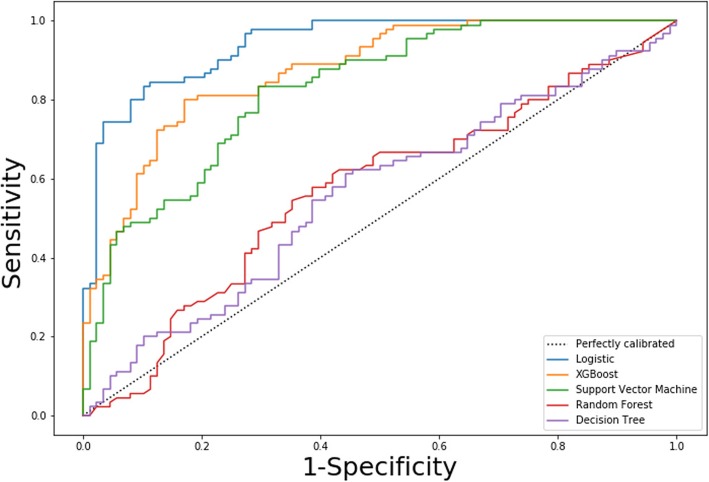
The receiver operating characteristic (ROC) curves of the multivariable logistic regression, random forest, support vector machine, X-Gradient boost, and decision tree demonstrate the determination of resectability of oesophageal squamous cell carcinoma in the validation cohort. XGboost = X-Gradient boost

The 8 radiomic features (all *P*-values < 0.01) were applied to develop the diagnostic model for identifying resectability of oesophageal SCC by using the training cohort. Five predictive models including the multivariable logistic regression, random forest, SVM, X-Gradient boost, and decision tree model were built. We selected the most suitable model from the 5 models depicted by AUC, accuracy, F-1score as shown in Table 3. The ROC curve (Fig. 4) indicated that the radiomic features that predicted resectability of oesophageal SCC were linearly separable. Therefore, the optimal radiomic features to predict resectability of oesophageal SCC was based on the multivariable logistic regression. Good performance of the radiomic model for the training cohort was observed for the multivariable logistic regression with an AUC of 0.92 ± 0.04, an accuracy of 0.87, and a F-1score of 0.93. This radiomic logistic model also showed good performance for predicting resectability of oesophageal SCC in the validation cohort (AUC, 0.87 ± 0.02; accuracy, 0.86; and F-1score, 0.86). The DeLong test showed that the multivariable logistic regression model had better performance than any other model for the identification of resectability of oesophageal SCC (all *P*-values < 0.001).

**Table 3:** Discrimination performance of radiomic features built by using the SVM, Decision tree, Random forest, X-Gradient boost and multivariable Logistic regression for the training and validation cohorts

Model	Discrimination		
	Accuracy	AUC ± SD	F-1score
The training cohort			
SVM	0.80	0.86 ± 0.03	0.81
Decision tree	0.69	0.73 ± 0.06	0.71
Random forest	0.73	0.80 ± 0.07	0.75
X-Gradient boost	0.78	0.87 ± 0.06	0.81
MLR	0.87	0.92 ± 0.04	0.93
The validation cohort			
SVM	0.79	0.82 ± 0.03	0.80
Decision tree	0.69	0.66 ± 0.03	0.70
Random forest	0.67	0.67 ± 0.03	0.68
X-Gradient boost	0.79	0.84 ± 0.03	0.79
MLR	0.86	0.87 ± 0.02	0.86

*SD* standard deviation, *SVM* support vector machine, *MLR* multivariable logistic regression, *AUC* receiver operating characteristic curve

**Fig. 5:**
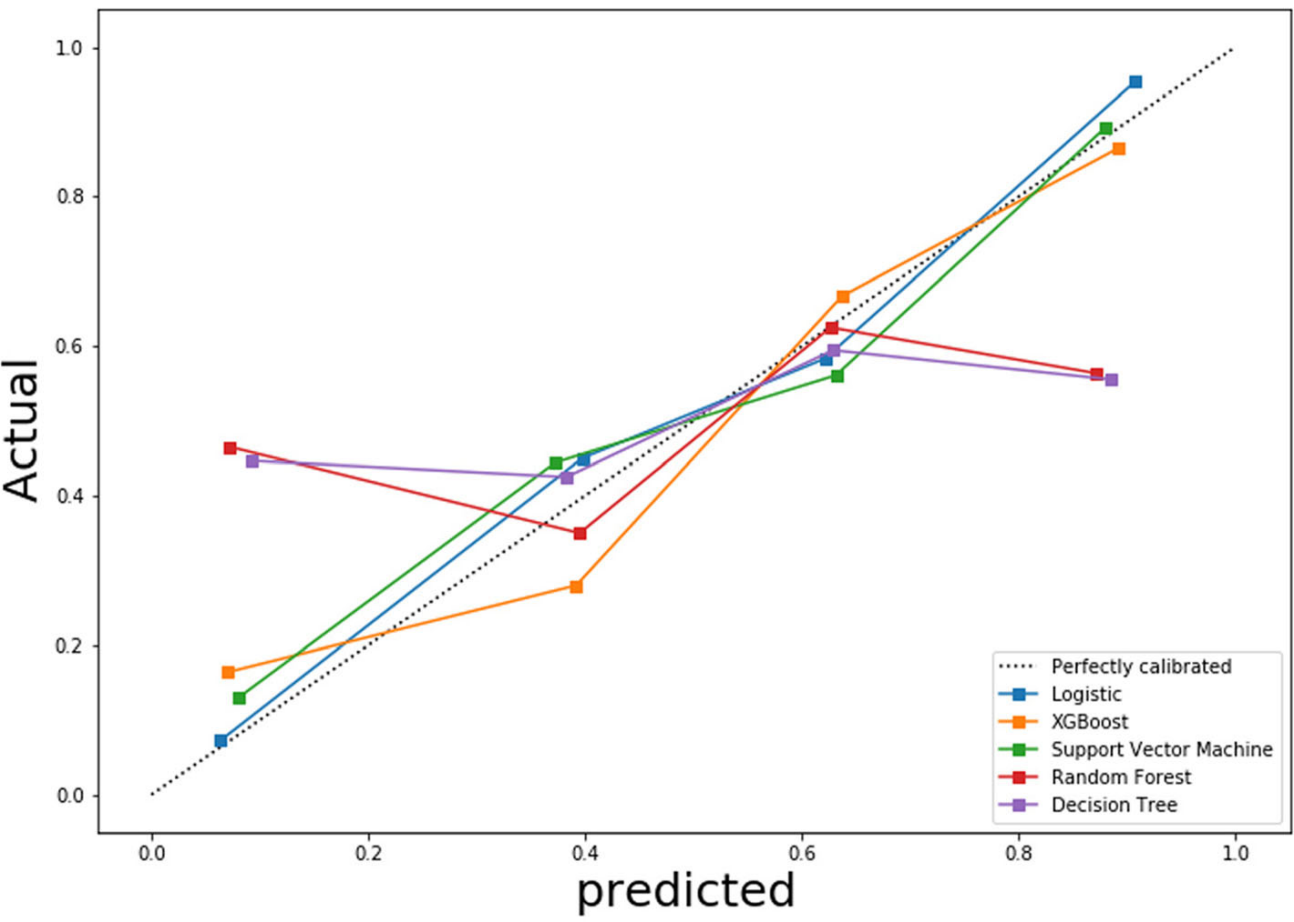
Calibration curves of the multivariable logistic regression, random forest, support vector machine, X-Gradient boost, and decision tree are for the prediction of resectability of oesophageal squamous cell carcinoma in the validation cohort. Actual and Predicted represent real and predicted oesophageal squamous cell carcinoma resection rates, respectively. XGboost = X-Gradient boost

In addition, good calibration was shown for the identification of resectability of oesophageal SCC in the validation cohort of the multivariable logistic regression model (*P* > 0.05) (Fig. 5). The Hosmer-Lemeshow test yielded a non-significant statistics (*P* > 0.05), which implied that there was no departure from perfect fit.

## Discussion

When compared to the basic CT features, radiomic features can objectively and quantitatively obtain more information about intratumour heterogeneity [5]. In this study, we developed and validated the CT radiomic features for the individualized identification of resectability of oesophageal SCC.

As shown in this study, 8 potential radiomic features including shape and intensity direct features, and texture features were selected from the 495 candidate features based on the contrast-enhanced CT data to build the CT radiomic model, which could be useful in assessing resectability of oesophageal SCC; and the 8 features of the 495 cases had a proper ratio for building this predicting model that could avoid overfitting. Of the 8 radiomic features, features of Convex and Orientation, which are shape and size features, describe the external contour information of the tumour, and they are highly consistent with the radiologists’ experience. The longer length and larger sphericity indicate more tumour invasions, hence, this leads to higher risk of resectability of oesophageal SCC. Although these two features can be captured subjectively, additional features can be extracted from CT images of oesophageal SCC, and these can be quantified and statistically analyzed. The five texture features and one intensity direct feature include X135.7Correlation, X45.1InverseVariance, X90.1InverseVariance, X90.1MaxProbability, Coarseness and Kurtosis, and mainly represent the texture complexity of tumours, which are highly associated with the tumours’ heterogeneity and prognosis [20, 24].

In the current study of the radiomic features identifying resectability of oesophageal SCC, a robust processing approach (10-fold cross-validation), which was feature reproducibility evaluation and wrapper-based feature selection as well as model establishment, was used to minimize the risk of modeling bias and over-fitting as reported by Paul et al. [13]. With these processes, the multivariable logistic regression model showed better performance than the random forest, support vector machine, X-Gradient boost or decision tree model, indicating sufficient discrimination. The possible reason for our findings may be that other models are too complex and are prone to over-fitting. The multivariable logistic regression model may help predict resectability of oesophageal SCC as a clinical adjunct tool for clinical treatment management.

There are several limitations in our study. First, we did not currently consider the genomic characteristics. To detect metastases in resected oesophageal SCC, the gene markers have attracted increasing attention in recent years, and some genes such as CXCR-2 and Cyclin D1 have been proposed in patients with oesophageal SCC [25, 26]. Radiogenomics is concerned with the relationship between imaging phenotypes and genomics. It has emerged in the field of tumour research and is attracting more and more attention. Although this may be a promising try, it is still to be considered whether establishing a radiomics model that utilizes the imaging features to predict results is superior to radiogenomic analysis [27]. Second, we lack of multicenter verification.

## Conclusions

Our study showed that CT radiomic features have the potential to predict resectability of oesophageal SCC especially in patients with initially unresectable oesophageal cancer who respond to neoadjuvant chemotherapy and changed to resectable tumours. The multivariable logistic regression model showed better performance than the random forest, support vector machine, X-Gradient boost or decision tree model to predict resectability of this cancer. We hope that our findings could be helpful for choosing the suitable treatment (surgical or other treatment) for oesophageal SCC patients to improve their survival rate.

## Supplementary information

**Additional file 1.** The extracted radiomic features.

## Data Availability

The data and material are available through the corresponding author (Dr. Tian-wu Chen).

## Abbreviations

3D: Three-dimensional
AUC: Area under the concentration-time curve
CT: Computed tomography
GLCM: Gray-level co-occurrence matrix
GLRLM: Gray-level run-length matrix
LASSO: Least absolute shrinkage and selection operator
NCCN: National Comprehensive Cancer Network
NGTDM: Neighborhood gray-tone difference matrix
ROC: Receiver operating characteristic
ROI: Region of interest
SCC: Squamous cell carcinoma
SVM: Support vector machine
