# Supplementary material for: CT radiomic features for predicting resectability of oesophageal squamous cell carcinoma as given by feature analysis: a case control study
Source: Cancer Imaging. 2019 Oct 16;19:66. doi: 10.1186/s40644-019-0254-0 (PMC6796480; doi:10.1186/s40644-019-0254-0)
Supplement: Supplementary file 1 — Additional file 1. The extracted radiomic features. [file 40644_2019_254_MOESM1_ESM.docx]

**Additional file 1**

**Category: Intensity Histogram**

| Variable | Description | Parameters |
| --- | --- | --- |
| Inter Quartile Range | The interquartile range of the occurence probability values in the histogram. | N/A |
| Kurtosis | Measure the peakedness of the occurence probability values in the histogram. | N/A |
| Mean Absolute Deviation | The mean absolute deviation of the occurence probability values in the histogram. | N/A |
| Median Absolute Deviation | The median absolute deviation of the occurence probability values in the histogram. | N/A |
| Percentile | Percentiles of the occurence probability values in the histogram. | Percentile=5 10 15 20 25 30 35 40 45 50 55 60 65 70 75 80 85 90 95; |
| Percentile Area | Percentiles of values in the accumulative histogram. | Percentile=5 10 15 20 25 30 35 40 45 50 55 60 65 70 75 80 85 90 95; |
| Quantile | Quantiles of the occurence probability values in the histogram. | Quantile=0.025 0.25 0.5 0.75 0.975; |
| Range | Measure the range (Max Value -Min Value) of the occurence probability values in the histogram. | N/A |
| Skewness | Measure the asymmetry of the occurence probability values in the histogram. | N/A |

**Category: Intensity Direct**

| Variable | Description | Parameters |
| --- | --- | --- |
| Energy (1) | N/A | N/A |
| Global Entropy | The intensity entropy among all the voxels. | N Bins=256; Range Min=0; Range Max=4096; Range Fix=1; |
| Global Max | The intensity maximum among all the voxels. | N/A |
| Global Mean | The intensity mean among all the voxels. | N/A |
| Global Median | The intensity median among all the voxels. | N/A |
| Global Min | The intensity minimum among all the voxels. | N/A |
| Global Std | The intensity standard deviation among all the voxels. | N/A |
| Global Uniformity | The intensity uniformity among all the voxels. | NBins=256; RangeMin=0; RangeMax=4096; RangeFix=1; |
| Inter Quartile Range | The interquartile range of the intensity values among all the voxels. | N/A |
| Kurtosis | Measure the peakedness of all the voxels' intensity. | N/A |
| Local Entropy Max | 1. First, at each voxel, compute entropy in its neighborhood region.  2. Then, compute the maximum among all the voxel's entropy caculated from 1. | NHood=9; RangeMin=0; RangeMax=4096; |
| Local Entropy Mean | 1. First, at each voxel, compute entropy in its neighborhood region.  2. Then, compute the mean among all the voxel's entropy caculated from 1. | NHood=9; RangeMin=0; RangeMax=4096; |
| Local Entropy Median | 1. First, at each voxel, compute entropy in its neighborhood region.  2. Then, compute the median among all the voxel's entropy caculated from 1. | NHood=9; RangeMin=0; RangeMax=4096; |
| Local Entropy Min | 1. First, at each voxel, compute entropy in its neighborhood region.  2. Then, compute the minimum among all the voxel's entropy caculated from 1. | NHood=9; RangeMin=0; RangeMax=4096; |
| Local Entropy Std | 1. First, at each voxel, compute entropy in its neighborhood region.  2. Then, compute the standard deviation among all the voxel's entropy caculated from 1. | NHood=9; RangeMin=0; RangeMax=4096; |
| Local Range Max | 1. First, at each voxel, compute range value(MaxValue-MinValue) in its neighborhood region.  2. Then, compute the median among all the voxel's range value caculated from 1. | NHood=5; |
| Local Range Mean | 1. First, at each voxel, compute range value(MaxValue-MinValue) in its neighborhood region.  2. Then, compute the mean among all the voxel's range value caculated from 1. | NHood=5; |
| Local Range Median | 1. First, at each voxel, compute range value (MaxValue - MinValue) in its neighborhood region.  2. Then, compute the median among all the voxel's range value caculated from 1. | NHood=5; |
| Local Range Min | 1. First, at each voxel, compute range value (MaxValue - MinValue) in its neighborhood region.  2. Then, compute the minimum among all the voxel's range value caculated from 1. | NHood=5; |
| Local Range Std | 1. First, at each voxel, compute range value (MaxValue - MinValue) in its neighborhood region.  2. Then, compute the standard deviation among all the voxel's range value caculated from 1. | NHood=5; |
| Local Std Max | 1. First, at each voxel, compute standard deviation in its neighborhood region.  2. Then, compute the maximum among all the voxel's standard deviation value caculated from 1. | NHood=5; |
| Local Std Mean | 1. First, at each voxel, compute standard deviation in its neighborhood region.  2. Then, compute the mean among all the voxel's standard deviation value caculated from 1. | NHood=5; |
| Local Std Median | 1. First, at each voxel, compute standard deviation in its neighborhood region.  2. Then, compute the median among all the voxel's standard deviation value caculated from 1. | NHood=5; |
| Local Std Min | 1. First, at each voxel, compute standard deviation in its neighborhood region.  2. Then, compute the mimimum among all the voxel's standard deviation value caculated from 1. | NHood=5; |
| Local Std Std | 1. First, at each voxel, compute standard deviation in its neighborhood region.  2. Then, compute the standard deviation all the voxel's standard deviation value caculated from 1. | NHood=5; |
| Mean Absolute Deviation | The mean absolute deviation of the intensity values among all the voxels. | N/A |
| Median Absolute Deviation | The median absolute deviation of the intensity values among all the voxels. | N/A |
| Percentile | Percentiles of the intensity values among all the voxels. | Percentile=5 10 15 20 25 30 35 40 45 50 55 60 65 70 75 80 85 90 95; |
| Quantile | Quantiles of the intensity values among all the voxels. | Quantile=0.025 0.25 0.5 0.75 0.975; |
| Range | The intensity range (Max Value - Min Value) among all the voxels. | N/A |
| Root Mean Square | N/A | N/A |
| Skewness | Measure the asymmetry of all the voxels' intensity. | N/A |
| Variance | N/A | N/A |

**Category: shape**

| Variable | Description | Formula | Parameters |
| --- | --- | --- | --- |
| Compactness 1 | N/A | (Volume)/(sqrt(pi)*(SurfaceArea)^(2/3)) | N/A |
| Compactness 2 | N/A | 36*pi*(Volume^2)/((SurfaceArea)^3) | N/A |
| Convex | 1. Measure the proportion of the pixels in the convex hull that are also in the region.  2. Refer to MATLAB "regionprops (Mask, 'Solidity')" for details | 1. Compute convex value in 2D slice-by-slice. Convex=regionprops (2DMask, 'Solidity')  2. Compute the mean of convex value among the slices. | N/A |
| Convex Hull Volume | The mean volume of the 2D convex hulls that are the convex envelopes of each slice's binary mask. | N/A | N/A |
| Convex Hull Volume3D | 3D volume of the convex hull that is the convex envelope of binary mask. | N/A | N/A |
| Mass | Mass caculation is only meaningful to CT images. | N/A | N/A |
| Max3D Diameter | Max3DDiameter= largest pairwise Euclidean distance between voxels on the surface of the tumor volume. | N/A | N/A |
| Mean Breadth | MeanBreath=Integral of mean curvature | N/A | N/A |
| Number of Objects | N/A | N/A | N/A |
| Number of Voxel | The number of voxels treating the edge voxels differently. | N/A | Edge Voxel Fraction=0.5; |
| Orientation | 1. Measure the angle between the x-axis and the major axis of the ellipse in 2D.  2. Refer to MATLAB "regionprops (Mask, 'Orientation')" for details | 1. First, compute orientation value in 2D slice-by-slice. Orientation=regionprops(2DMask, 'Orientation')  2. Then, compute the mean of orientation value among the slices. | N/A |
| Roundness | 1. Measure how much the binary mask is close to circle in 2D.  2. Refer to MATLAB "regionprops(Mask, 'Eccentricity')" for details | 1. First, compute roundness value in 2D slice-by-slice. Roundness=1-regionprops(2DMask, 'Eccentricity')  2. Then, compute the mean of roundness value among the slices. | N/A |
| Spherical Disproportion | N/A | N/A | N/A |
| Sphericity | N/A | N/A | N/A |
| Surface Area | The surface area of the binary mask. | N/A | N/A |
| Surface Area Density | Surface Area Density == Surface Volume Ration in Hugo's paper below. | (surface area of the binary mask)/(volume of the binary mask). | N/A |
| Volume | The physical volume treating the edge voxels differently. | N/A | Edge Voxel Fraction=0.5; |
| Voxel Size | The physical voxel size. | N/A | N/A |

**Category: Gray Level Cooccurence Matrix 25**

| Variable | Description |
| --- | --- |
| Auto Correlation | N/A |
| Cluster Prominen | N/A |
| Cluster Shade | N/A |
| Cluster Tendendcy | N/A |
| Contrast | For the feature description, refer to the documentation on MATLAB function "gray co-props". |
| Correlation | For the feature description, refer to the documentation on MATLAB function "gray co-props". |
| Difference Entropy | N/A |
| Dissimilarity |  |
| Energy | For the feature description, refer to the documentation on MATLAB function "graycoprops". |
| Entropy |  |
| Homogeneity | 1.This feature is equivalent to Homogeneity1 in Hugo's paper.  2. For the feature description, refer to the documentation on MATLAB function "gray co-props". |
| Homogeneity2 | N/A |
| InformationMeasureCorr1 | N/A |
| InformationMeasureCorr2 | N/A |
| Inverse Diff Moment Norm | N/A |
| Inverse Diff Norm | N/A |
| Inverse Variance | N/A |
| Max Probability | N/A |
| Sum Average | N/A |
| Sum Entropy | N/A |
| Sum Variance | N/A |
| Variance | N/A |

**Category: Gray Level Run Length Matrix 25**

| Variable |
| --- |
| Gray Level Non uniformity |
| High Gray Level Run Empha |
| Long Run Emphasis |
| Long Run High Gray Level Empha |
| Long Run Low Gray Level Empha |
| Low Gray Level Run Empha |
| Run Length Non uniformity |
| Run Percentage |
| Short Run Emphasis |
| Short Run High Gray Level Empha |
| Short Run Low Gray Level Empha |

**Category: Neighbor Intensity Difference 25**

| Variable |
| --- |
| Busyness |
| Coarseness |
| Complexity |
| Contrast |
| Texture Strength |
